# Hippocampal volume varies with educational attainment across the life-span

**DOI:** 10.3389/fnhum.2012.00307

**Published:** 2012-11-09

**Authors:** Kimberly G. Noble, Stuart M. Grieve, Mayuresh S. Korgaonkar, Laura E. Engelhardt, Erica Y. Griffith, Leanne M. Williams, Adam M. Brickman

**Affiliations:** ^1^Department of Pediatrics, College of Physicians and Surgeons, Columbia UniversityNew York, NY, USA; ^2^Gertrude H. Sergievsky Center, College of Physicians and Surgeons, Columbia UniversityNew York, NY, USA; ^3^Brain Dynamics Centre, University of Sydney Medical School and Westmead Millennium InstituteSydney, NSW, Australia; ^4^Taub Institute for Research on Alzheimer's Disease and the Aging Brain, College of Physicians and Surgeons, Columbia UniversityNew York, NY, USA; ^5^Department of Neurology, College of Physicians and Surgeons, Columbia UniversityNew York, NY, USA

**Keywords:** socioeconomic status, SES, education, hippocampus, amygdala, brain, neuroanatomy, brain reserve

## Abstract

Socioeconomic disparities—and particularly differences in educational attainment—are associated with remarkable differences in cognition and behavior across the life-span. Decreased educational attainment has been linked to increased exposure to life stressors, which in turn have been associated with structural differences in the hippocampus and the amygdala. However, the degree to which educational attainment is directly associated with anatomical differences in these structures remains unclear. Recent studies in children have found socioeconomic differences in regional brain volume in the hippocampus and amygdala across childhood and adolescence. Here we expand on this work, by investigating whether disparities in hippocampal and amygdala volume persist across the life-span. In a sample of 275 individuals from the BRAINnet Foundation database ranging in age from 17 to 87, we found that socioeconomic status (SES), as operationalized by years of educational attainment, moderates the effect of age on hippocampal volume. Specifically, hippocampal volume tended to markedly decrease with age among less educated individuals, whereas age-related reductions in hippocampal volume were less pronounced among more highly educated individuals. No such effects were found for amygdala volume. Possible mechanisms by which education may buffer age-related effects on hippocampal volume are discussed.

## Introduction

Socioeconomic disparities are associated with remarkable differences in cognition and behavior (Noble et al., 2005, 2007; Farah et al., 2006; D'Angiulli et al., 2008; Gianaros et al., 2008; Hackman and Farah, 2009; Kishiyama et al., 2009; Stevens et al., 2009; Raizada and Kishiyama, 2010), with ramifications for physical and mental health across the lifespan (Brooks-Gunn and Duncan, 1997; McLoyd, 1998; Gianaros and Manuck, 2010).

Socioeconomic status (SES) is typically characterized by multiple factors, including educational attainment, occupation, and income level (McLoyd, 1998). In childhood, SES is typically quantified by measuring parental levels of these indicators. In contrast, studies of SES among adults more commonly focus on an individual's *own* socioeconomic position (Stern, 2002; Scarmeas et al., 2006; Gianaros et al., 2007a). Adult socioeconomic attainment may influence cognition across the life-course independently of childhood SES (Turrell et al., 2002), and the effects of childhood SES may operate on adult cognitive achievement indirectly through adult socioeconomic position (Singh-Manoux et al., 2005).

Different components of SES may operate differentially on specific life outcomes, via different mechanisms (Duncan and Magnuson, 2012; Noble et al., 2012). However, across the lifespan, educational attainment is arguably the single socioeconomic variable that best predicts both cognitive and neural outcomes. For example, in adulthood, an individual's level of education has been related to an array of cognitive skills, including processing speed, working memory, verbal fluency, and episodic memory (Zahodne et al., 2011). Similarly, in healthy elderly individuals, some investigators have reported associations between higher education and increased cortical thickness across a large number of cortical regions (Liu et al., 2012) although other studies have failed to find similar relationships (Seo et al., 2011).

Educational attainment may have differential effects on particular brain regions over the life course. Of course, SES in general, and educational attainment specifically, is a marker for a broad conglomerate of experiences and exposures. Many environmental factors have been shown to affect regionally specific brain development (Rosenzweig, 2003; McEwen and Gianaros, 2010), and thus are likely candidates in mediating the links between educational disparities and specific neurocognitive outcomes. For example, lower levels of education have been associated with increased exposure to numerous life stressors, including uncertainty about material resources such as food or clothing; chaotic households; and exposure to violence (Evans, 2004). Exposure to stressors may in turn relate to structural differences in specific brain regions that are particularly responsive to stress, including the hippocampus (Buss et al., 2007; Felmingham et al., 2009; McEwen and Gianaros, 2010; Tottenham and Sheridan, 2010 Teicher et al., 2012) and the amygdala (McEwen and Gianaros, 2010; Tottenham and Sheridan, 2010). However, results have been mixed concerning the degree to which educational attainment is itself directly associated with measurable anatomic differences in these two neural regions.

One recent study found that higher educational attainment was associated with decreased white matter mean diffusivity in the hippocampus (Piras et al., 2011). However, other studies have failed to find effects of educational attainment on hippocampal volume (Kidron et al., 1997; Hanson et al., 2011; Noble et al., 2012) or cortical thickness (Liu et al., 2012). One recent study found that childhood SES, as operationalized by adult recall of childhood home conditions and paternal occupation, predicted adult hippocampal volume, whereas adult educational attainment did not (Staff et al., 2012). Further, one study of postmenopausal women found that, although hippocampal volume varied as a function of perceived stress, this effect was not accounted for by differences in education levels (Gianaros et al., 2007b).

In the amygdala, some investigators have reported positive relationships between educational attainment and regional volume (Laakso et al., 1995; Noble et al., 2012) while others have not (Gianaros et al., 2007b; Hanson et al., 2011). Other studies have reported significant associations between levels of education (Dannlowski et al., 2012) or subjective social status (Gianaros et al., 2008; Muscatell et al., 2012) and amygdala function.

One way to reconcile these seemingly disparate findings is to note that, although a number studies have examined the effects of educational disparities on regional brain structure, the majority have done so within a relatively narrow age range. To our knowledge, no study has assessed whether any effect of educational attainment on these structures may be moderated by the age of participants across the lifespan. As the absence of a main effect is uninterpretable in the presence of an interaction (Pedhazur, 1997a), this represents a significant gap in the literature.

The theory of “brain reserve” suggests that higher levels of education may confer benefits in brain structure or function that may buffer against age-related changes (Stern, 2002, 2006). Early exposure to cognitively stimulating experiences may be protective against later insult (Holt and Mikati, 2011). For example, one recent study found that, while greater educational attainment did not protect individuals from neuropathology, it did reduce the risk of developing clinical dementia relative to less educated individuals with similar degrees of pathology (Brayne et al., 2010). In that study, education was also associated with brain weight, suggesting that the compensatory influence of education may be mediated by increased regional or global brain volume.

Based on this reasoning, we hypothesized that educational attainment may be associated with regional volumetric differences in the hippocampus and amygdala across the life-course. Further, we predicted that the effects of educational attainment would not be consistent across all ages, but rather would be most pronounced in buffering against age-related decline. To test these hypotheses, we employed structural magnetic resonance imaging (MRI) to assess regional hippocampal and amygdala volume in an educationally diverse sample of adults across a seven-decade age range.

## Materials and methods

### Subjects

Subjects were compiled from the Brain Resource International Database, accessed via the independently governed 501(c)(3) BRAINnet Foundation (http://www.BRAINnet.net). Wave I data from this standardized database comprise demographic, self-report, physiologic, and neuroimaging data on healthy participants ranging in age from 7 to 87, collected from six primary sites throughout the world (Gordon et al., 2005; Grieve et al., 2005). For the current study, participants included the 275 individuals (152 female) who were 17 years of age or older (average age 39.7 years, s.d. 17, range 17–87), and for whom both years of educational attainment as well as hippocampal and amygdala regional volumes were available (see Table 1). The majority of the sample self-identified as having European ancestry (74%). Additionally, 5.1% of the sample self-identified as having African ancestry, 5.8% were of Asian ancestry (including Middle Eastern or Indian subcontinent), 0.4% were Indigenous Australian, 0.4% were Pacific Islander, 8.4% identified as mixed race/ethnicity, and 5.8% preferred not to answer.

**Table 1:** **Demographics of sample**.

**Education level**	**High school or less**	**Some college**	**College and up**	**Total**
*N*	89	83	103	275
Age: Mean (s.d.)	48 (19)	35 (16)	36 (13)	39.7 (17)
Sex	49 female, 40 male	42 female, 41 male	83 female, 89 male	152 female, 123 male

This participant cohort represents a community sample found in the major cities of Australia with no known bias in region-wise education or distribution of educational attainment. Although individuals from lower socioeconomic backgrounds are often difficult to recruit to participate in research studies, educational attainment of the sample spanned a wide range (mean 14 years, s.d. 3.1, range 3–18 years). This range of education is due in part to the fact that, across Australia, educational attainment is fairly diverse, with approximately 25% of individuals not earning a high school diploma, approximately 45% of individuals completing high school only, and approximately 30% completing some type of post-secondary education (Pink and Australian Bureau of Statistics, 2010).

MRI data sets came from two imaging sites: Westmead Hospital (Sydney, Australia) and Wakefield Imaging (Adelaide, Australia). All participants in the study were recruited from the general population in the Sydney and Adelaide urban and suburban areas within Australia through standard recruitment procedures (e.g., community advertisements). Cross-site reliability has been established (Grieve et al., 2005). As age and educational attainment are nearly perfectly correlated in younger children, children in the database under 17 years old were excluded from the analysis, as it would be impossible to de-confound the effects of age and education among this group. By the late teenage years, however, it is possible to distinguish the effects of age and education. For example, among the 35 teenagers in the sample aged 17–19, the range of education spanned from 11 (less than high school) to 16 years (college degree). This is possible, in part, because not all individuals complete high school and/or go on to college, and conversely, because most Australian universities have mechanisms for accepting talented youngsters to university early, before the completion of high school (Victorian Government Department of Education and Early Childhood Development, 2010).

Participants completed WebQ, a standardized computer-based battery of questionnaires that assess medical history, demographics, and psychological function, including current or lifetime diagnosis of neurological and psychiatric (Axis 1) conditions (Heatherton et al., 1991; Trzepacz and Baker, 1993; Bush et al., 1998; Spitzer et al., 1999; Breslau and Kessler, 2001; Hickie et al., 2001) (for more information see Gatt et al., 2009; Williams et al., 2009). All participants in the database were excluded from further participation if they had history of brain injury, significant medical, neurological or psychiatric conditions, and/or drug or alcohol addiction. Individuals with first-degree family members with an Axis I disorder were also excluded. No specific screening for neurodegenerative disorders was performed. However, a subset of participants completed a test of memory performance, as described below.

All participants provided informed consent in accordance with the National Health and Medical Research Council of Australia guidelines. Participation was voluntary and participants were reimbursed to cover cost for their time and travel to the research center.

### Memory testing

One hundred fourteen of the participants had complete data on a Memory Recall and Recognition test. This test is a variant of the Rey Auditory Verbal Learning and Memory task (Rey, 1964; Geffen et al., 1990), commonly used to provide measures of auditory-verbal learning, recall, and recognition. Briefly, the participant is presented with a sequence of 12 words binaurally via headphones, 1 s at a time. The participant is instructed to say back as many of the words as they can remember from the list, in any order. This procedure is then repeated three more times, with the same instructions. The total number of words recalled during these four trials is recorded. The participant is then presented with a second list of words (which are neither semantically nor phonetically related to the first list) and asked to say back as many of these as can be remembered (Distractor Trial). Immediately following this trial, the participant is asked to recall as many words as can be remembered from the first list (Immediate Recall Trial). Approximately 25 min later, after completing a number of other tests in the battery, the participant is again asked to recall as many words as possible from the first list (Delayed Recall Trial). The subject is then presented one at a time with a series of 24 words on the computer screen (Recognition Trial). Half of these words are the words from the first list; the remaining words are new words. The words are in fixed, pseudorandom order. Following each word, the participant is required to touch a “Yes” or “No” button on the touchscreen according to whether or not the word was in the first list.

### MRI scan acquisition

MRI was conducted on a 1.5 Tesla Siemens Sonata system at Westmead Hospital, Sydney and a 1.5 Tesla Siemens Sonata at Perrett Imaging, Flinders University, Australia. The MRI protocol included a 3-D T1-weighted image acquired in the sagittal plane using an MPRAGE sequence (TR = 9.7 ms; TE = 4 ms; echo train 7; flip angle = 12°; TI = 200 ms; NEX = 1). A total of 180 1 mm slices (without gap) were acquired with a 256 × 256 matrix with an in plane resolution of 1 mm × 1 mm. Approximately 1% of the MRI data were excluded from the overall database due to MRI technical acquisition errors/artifact.

### MRI analysis

**Figure 1:**
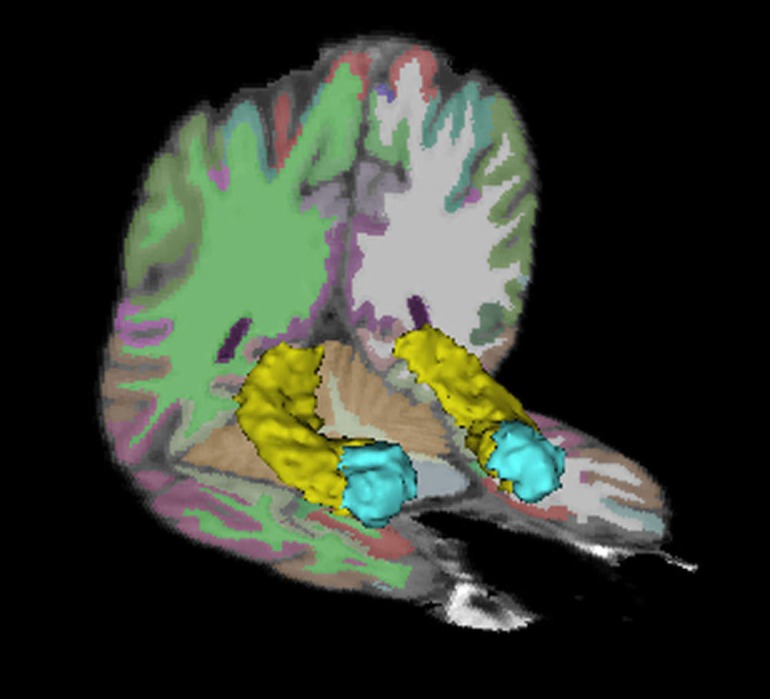
**Hippocampus and amygdala regions of interest.** The bilateral hippocampi are depicted in yellow. The bilateral amygdalae are depicted in blue.

Volumetric segmentation was performed on the 3D T1-weighted structural images with the FreeSurfer image analysis suite (version 4.3) (http://surfer.nmr.mgh.harvard.edu/), which has been shown to be comparable in accuracy to manual labeling (Fischl et al., 2002). The technical details of these procedures are described in detail elsewhere (Dale et al., 1999; Fischl et al., 1999; Fisher and Defries, 2002; Grieve et al., 2011). Volumetric measurements of the hippocampus and the amygdala were generated with FreeSurfer's automatic quantification of subcortical structures, which assigns a neuroanatomical label to each voxel in an MRI volume based on probabilistic information estimated from a manually labeled training set (see Figure 1). The classification technique employs a non-linear registration procedure that is robust to anatomical variability, as described in detail elsewhere (Fischl et al., 2002). Anatomically labeled structures were manually inspected for accuracy, and corrected if necessary. Total brain volumes, which were used as a covariate in the statistical analyses, were also generated automatically with the FreeSurfer processing stream.

### Statistical analyses

To assess whether educational attainment influences regional brain volume across the life-span, we conducted a series of regression analyses, with total hippocampal volume or amygdala volume as the dependent variable, and years of educational attainment as the independent variable of interest. Analyses included age, sex, and total brain volume as covariates. We hypothesized that educational attainment would account for variation in regional brain volume, adjusting for age, sex, and total brain volume. Additionally, we examined the extent to which age-related regional brain volumetric differences would be modified by educational attainment. Specifically, we predicted a significant education × age interaction, such that age-related decreases in regional brain volume would be less steep among more highly educated individuals.

For ease of presentation, results in tables and figures are at times presented using categorical bins of age and educational attainment. However, unless otherwise noted, statistical analyses considered age in years and years of education as continuous variables, to take advantage of the full level of detail provided by the dataset.

## Results

### Regional volumetric data

Table 2 shows hippocampal and amygdala volumetric data, broken down by age and gender categories.

**Table 2:** **Hippocampus and amygdala volume, by age and sex**.

	**Hippocampal volume, mm^3^ mean (s.d.)**	**Amygdala volume, mm^3^ mean (s.d.)**
Males <35	9004 (871)	3421 (352)
Males 35 and up	8187 (1063)	3103 (455)
Females <35	8248 (711)	2968 (306)
Females 35 and up	7893 (830)	2847 (329)

Both hippocampal and amygdala volumes were normally distributed across the sample, without outliers (Hippocampus: skewness = −0.089 (SE 0.147), kurtosis = 1.175 (SE 0.293), Kolmogorov–Smirnov normality statistic = 0.041, *p* = 0.2; Amygdala: skewness = 0.306 (SE 0.147), kurtosis = 0.618, (SE 0.293), Kolmogorov–Smirnov normality statistic = 0.041, *p* = 0.2).

### Main effects—hippocampus

Initial bivariate correlations showed that age was significantly negatively associated with hippocampal size across the entire age range (*r* = −0.374; *p* < 2.1 × 10^−11^). However, the data were better fit by a quadratic function for age (*r* = −0.402; *p* < 4.3 × 10^−12^), reflecting the fact that hippocampal volume tends to increase until approximately age 30, at which point hippocampal volume tends to decline (Grieve et al., 2011). Age^2^ was therefore included as a regressor in all models below.

Additionally, across the entire age range, educational attainment was positively and linearly associated with hippocampal size (*R* = 0.236; *p* < 7.9 × 10^−5^). When adjusting for age, sex, and total brain volume, however, the main effect of educational attainment did not account for significant unique variance in total hippocampal volume (Beta = 0.078; *p* < 0.076), left hippocampal volume (Beta = 0.086; *p* < 0.053), or right hippocampal volume (Beta = 0.065; *p* < 0.15).

### Main effects—amygdala

Similar to the findings in the hippocampus, bivariate correlations revealed a significant negative association between amygdala volume and age (*r* = −0.239; *p* < 2.9 × 10^−5^), with the data best fit by a quadratic function for age (*r* = −0.254; *p* < 2.04 × 10^−5^) (Grieve et al., 2011).

A significant linear association between amygdala volume and educational attainment was also observed (*r* = 0.154; *p* < 0.01). Again, however, the main effect of educational attainment on total amygdala volume was no longer significant when adjusting for age, sex, and total brain volume (Beta = 0.029; *p* = 0.515). Nor was this effect significant when the left (Beta = 0.073; *p* < 0.094) or right (Beta = −0.016; *p* < 0.744) amygdala were considered separately.

### Age by education interactions

**Figure 2:**
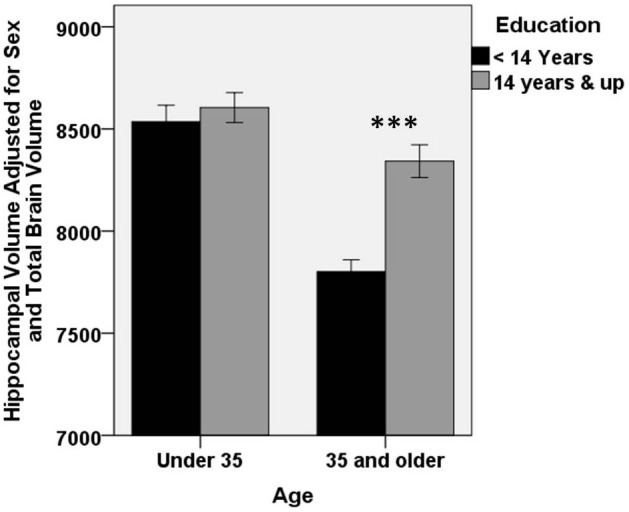
**Higher educational attainment buffers age-related decrease in hippocampal volume.** A significant education × age interaction is present for hippocampal volume (Beta = 0.498 *p* < 0.001), such that age-related decline in hippocampal volume is smaller among individuals with higher educational attainment. For clarity of presentation, educational attainment and age are represented graphically by median split. There was no difference in hippocampal volume according to education in the younger half of the subject pool. However, among older participants, more highly educated individuals had significantly larger hippocampi (^***^p < 0.0001). Error bars represent ±1 standard error.

Of interest next was the extent to which the effect of age on regional volume varied by an individual's educational attainment. To assess this hypothesis, models above were extended to include terms for an age × education interaction. We first assessed the hippocampus. A significant age × education interaction was present (*R*^2^ change = 0.021, Beta = 0.498 *p* < 0.001), suggesting that the effect of age on hippocampal volume is not constant across all levels of educational attainment. This finding is portrayed in Figure 2, where it can be seen that the age-related decline in hippocampal volume is moderated by education. In this figure, the data are divided according to median split of individuals by age and education. There is no difference in hippocampal size by education among individuals younger than 35 years of age [*t*_(136)_ = −0.636; *p* = 0.53]. However, among individuals older than 35 years, there is a highly significant difference in hippocampal size, with more highly educated individuals having larger hippocampi [*t*_(135)_ = −5.6; *p* < 0.0001].

When considered separately, both left and right hippocampi showed a similar interaction (left hippocampus: Beta = 0.508, *p* < 0.001; right hippocampus: Beta = 0.462, *p* < 0.002).

**Figure 3:**
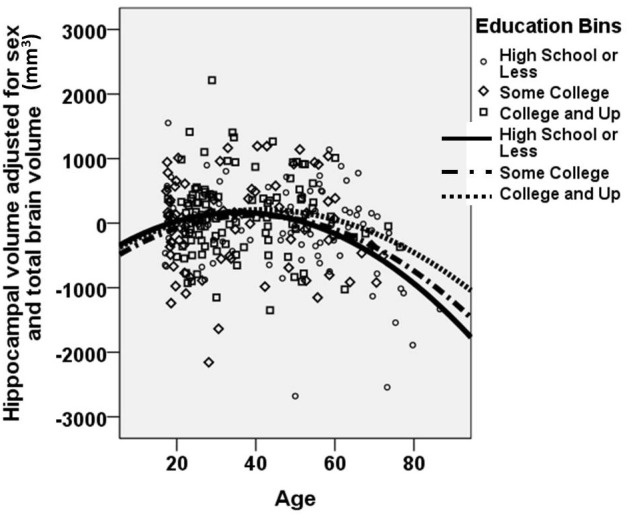
**A dose-dependent moderating effect of education on age-related decrease in hippocampal volume.** A “dose-dependent” effect of education is observed. Age-related decline in hippocampal volume is most pronounced among the least educated individuals. In contrast, age-related reduction in hippocampal volume is mitigated among individuals with higher educational attainment. Hippocampal volume is portrayed as the unstandardized residual, after adjusting for sex and total brain volume. The figure excludes the 13 data points that represented “influential points” as identified using the standardized difference in beta. Note that all analyses were performed using continuous variables for age, years of educational attainment, and hippocampal volume. For clarity of presentation, educational attainment is represented graphically in bins, with high school or less portrayed with circles/solid line; some college portrayed with diamonds/dashed-dotted line; and college or beyond portrayed with squares/dotted line.

Figure 3 shows a “dose-dependent” effect of education: The observed age-related volumetric decreases in hippocampal volume are greater among less educated individuals relative to more highly educated individuals. Less difference in hippocampal volume is seen with age among the highest educated individuals.

Of note, there was a wide span of educational attainment across the entire age range, with the educational spectrum represented across each age range, from young adults to elderly individuals (see Table 1). Nonetheless, there was a significant correlation between age and education (*r* = −0.257; *p* < 0.0001), such that older participants were on average less educated than younger participants. For this reason, we also examined the full model excluding “influential points” in this correlation. Influential points were identified using the standardized difference in beta, defined as the difference in the standardized regression coefficient as a result of deleting a particular case, divided by the standard error of this difference. Any value greater than 2/√n was excluded (Pedhazur, 1997b). Among the remaining 267 participants, the age × education interaction remained significant (Beta = 0.454; *p* < 0.001), suggesting that the moderating effect of education on age-related decreases in hippocampal volume is an accurate description of the sample, and not a spurious finding driven by the influence of a small number of subjects with extreme values. These 267 participants are represented in Figure 3.

No significant education × age interaction was observed in total amygdala volume, or left or right hemispheres considered separately, regardless of whether influential points were included or excluded, suggesting that the effect of age on amygdala volume is independent of educational attainment.

### Memory testing

No specific clinical testing was done to assess whether participants may have had early signs of a neurodegenerative disease. However, the scores of the 114 participants who had valid data for the Memory Recall and Recognition test were extremely similar to those of the 1007 participants in the full BrainNet International Database, from which our sample was drawn (Clark et al., 2006), (see Table 3).

**Table 3:** **Memory task performance in the present sample relative to the full database**.

		**Males**		**Females**	
		**Mean**	**s.d.**	**Mean**	**s.d.**
Immediate	Present sample	32.13	4.31	33.19	4.80
recall	(*n* = 114)				
	Full database	30.76	6.98	31.78	7.17
Short delay	Present sample	7.96	1.96	8.41	1.88
recall	(*n* = 114)				
	Full database	7.52	2.62	8.04	2.60
Long delay	Present sample	7.41	1.93	7.90	2.17
recall	(*n* = 114)				
	Full database	7.34	2.40	7.89	2.52
Recognition	Present sample	11.16	1.04	11.14	1.57
	(*n* = 114)				
	Full database	10.96	1.40	11.06	1.30

On the total immediate recall score, 100% of participants in the present study performed within one standard deviation of the full sample mean. On the short delay score, 98% of participants scored within one standard deviation of the full sample mean, and 100% scored within two standard deviations. On the long delay score, 95% of participants scored within one standard deviation, and 98% scored within two standard deviations of the full sample mean. On the recognition accuracy score, 96% scored within one standard deviation, and 98% scored within two standard deviations of the full sample mean. When excluding participants who scored more than two standard deviations below the database mean on any memory subtest, the age × education interaction in hippocampal volume remained significant (Beta = 0.504; *p* < 0.0004).

## Discussion

Here we found that educational attainment buffers against age-related differences in hippocampal volume. Similar results were not found in the amygdala, a nearby medial temporal lobe structure, suggesting that results may be at least somewhat specific to the hippocampus.

### Implications

The idea that, among older individuals, higher levels of education confer benefits in brain structure or function that buffer against age-related changes or brain pathology is often referred to as “brain” or “cognitive” reserve (Stern, 2002, 2006). For instance, some studies have found that Alzheimer's Disease (AD) is less prevalent among individuals with higher levels of education (Kidron et al., 1997), and it has been suggested that this may be due to a protective effect whereby individuals with more education may not manifest clinical symptoms of disease until higher levels of neural pathology are reached (Scarmeas et al., 2006; Julkunen et al., 2010; Seo et al., 2011; Liu et al., 2012).

The present study provides some support for this theory. A “dose-dependent” effect of education was found, such that age-related decreases in hippocampal volume were steepest among the least educated individuals. In contrast, age-related decrease in hippocampal volume was less pronounced among individuals with higher educational attainment. It is thus possible that, among this sample of healthy individuals, higher levels of education were able to counteract typical age-related changes in hippocampal structure. The functional significance of these differential rates of decline may reflect greater retention of the cognitive functions, including declarative memory performance, which rely on this structure (Grieve et al., 2011).

### Possible mechanisms

Many components of SES tend to vary with educational attainment and may themselves be important factors in understanding mechanistic pathways. Other objective socioeconomic indicators include measures of income and occupation, which were not available in the dataset studied here. Although education, occupation and income tend to be highly correlated (McLoyd, 1998), they may nonetheless act independently in predicting different outcomes of interest (Duncan and Magnuson, 2012; Noble et al., 2012). For instance, previous work has suggested that family income levels and parental educational attainment may not act in concert in predicting hippocampal and amygdala volumes in children (Hanson et al., 2011; Noble et al., 2012). Further, subjective social status, or an individual's perception of where he or she stands in the social hierarchy, may predict regional brain structure and/or function over and above objective socioeconomic measures (Gianaros et al., 2007a, 2008).

Socioeconomic variables—including differences in educational attainment—likely represent relatively distal forces operating on cognitive and neural outcomes. However, SES has been associated with many factors that have been shown to exert more proximal effects on regional brain structure. One well described pathway includes differences in exposure to stress or allostatic load (McEwen, 1998, 1999, 2000, 2001a,b; Evans, 2004; Evans and Schamberg, 2009; Gianaros and Manuck, 2010; McEwen and Gianaros, 2010).

In both animals and humans, the experience of stress has been linked to differences in the structure of the hippocampus (Rao et al., 2010; Tottenham and Sheridan, 2010). The animal literature has shown cascading negative effects of early life stress on hippocampal development at the cellular, anatomic and functional levels (Sapolsky, 1996; McEwen, 1999; Brunson et al., 2005; Gianaros and Manuck, 2010; McEwen and Gianaros, 2010; Tottenham and Sheridan, 2010). Conversely, adult offspring of female rodents that exhibit high licking and grooming of pups—a model of “parental nurturance”—show increased hippocampal synaptic density and plasticity (Liu et al., 2000; Champagne et al., 2008), and improved performance in hippocampal-dependent forms of memory (Liu et al., 2000).

In studies of human adults, self-reported stress exposure, stress-related mental illness, and the prior experience of child abuse have all been associated with decreased hippocampal volume (Sheline et al., 1996; Bremner et al., 1997; Geuze et al., 2005; Kitayama et al., 2005; Gianaros et al., 2007b; Tottenham and Sheridan, 2010; Dannlowski et al., 2012). [Findings concerning the association between stress exposure and hippocampal size in children, however, have been less consistent (Woon and Hedges, 2008; De Bellis et al., 2010; Rao et al., 2010)].

In the socioeconomically diverse sample of adults studied here, one possibility is that differences in the experience of stress may mediate the association between educational attainment and hippocampal volume. Future research will be necessary to directly test this putative mediating pathway.

Interestingly, and contrary to initial predictions, we only found a moderating effect of educational attainment on regional volume in the hippocampus, and not in the amygdala. Research in animals has demonstrated that the experience of stress profoundly affects development of the amygdala (Makino et al., 1994, 1999; Bonaz and Rivest, 1998; Wood et al., 2003; Moriceau, 2004), and human neuroimaging studies also suggest that the amygdala may be structurally altered by stress (Tottenham et al., 2010). Thus, if the reported effects of education could be completely explained by differences in exposure to stress, one would have expected to see effects in the amygdala as well. It is therefore likely that some other mechanistic factors might explain the effects of educational attainment on hippocampal volume described here. Of course, many factors, including but not limited to prenatal factors, nutrition, exercise, parenting, and the quality and quantity of the schooling experience, are associated with an individual's educational attainment, and may serve as more proximal mechanisms underlying the results reported here. Indeed, it is likely that multiple such experiential factors contribute to the moderating effects of educational attainment on hippocampal volume.

Finally, while differences in experience have direct effects on the hippocampus, it should also be noted that interactions between age, educational attainment and hippocampal volume may in part be explained by differences in genes and/or gene-environment interactions. Future research will be necessary to disentangle the various causal pathways.

### Effects of education across the lifespan

In a recent study of 60 socioeconomically diverse children and adolescents, we found that parental education levels predicted children's amygdala volumes, but not hippocampal volumes (Noble et al., 2012). (In contrast, parental income levels predicted hippocampal but not amygdala volumes.) This differs from the findings reported here, in which educational attainment moderated hippocampal, but not amygdala volume. Several notable differences may help to reconcile these findings.

The two studies included a non-overlapping age range of participants: the previous study examined children between the ages of 5 and 17, whereas the present study includes individuals between the ages of 17 and 87. Human neuroimaging studies have suggested that the effects of adversity on amygdala structure may vary based on the age of the population studied. In adulthood, the experience of highly stressful events has been associated with smaller amygdala size (Driessen et al., 2000; Schmahl et al., 2003; Tottenham and Sheridan, 2010), whereas in children, adverse caregiving circumstances have been associated with atypically large amygdala volume (Mehta et al., 2009; Tottenham et al., 2010). Thus, it is similarly possible that any effects of education on amygdala structure may be different in childhood versus adulthood, even considering the broad range of adult ages studied here.

A second possible explanation is that the previous study in children examined *parental* education levels, whereas the current study examined the *participants'* education levels. (Unfortunately parental educational attainment was not available in the current dataset.) It is therefore possible that differences in parental education may reflect differences in childhood experience or parenting that are not necessarily captured by one's own ultimate educational attainment. For instance, lower parent education has been associated with lower levels of parental nurturance (Brooks-Gunn and Markman, 2005), which may have particular importance for amygdala structure (Tottenham et al., 2010). Additionally, it is possible that the association between adult educational attainment and hippocampal volume may be mediated by the material resources individuals were exposed to as children (e.g., parental SES). Indeed, differential effects on hippocampal volumes have been described as a function of childhood SES versus adult SES. For example, several reports have found that higher childhood SES is associated with larger hippocampi (Hanson et al., 2011; Jednoróg et al., 2012; Noble et al., 2012). Intriguingly, Staff et al. (2012) recently reported that childhood SES, as operationalized by adult recall of childhood home conditions and paternal occupation, predicted adult hippocampal volume, whereas adult SES did not. This suggests that the high degree of neural plasticity in childhood may underlie effects observed decades later. Ideally, future work in adults will incorporate measures of both parental education and participant education to better sort out these disparate effects.

A third possibility is that the previous study, with only 60 participants, had reduced power with which to detect an effect of education on hippocampal volume, whereas the greater sample size in the present study allowed such an effect to be revealed here. This explanation alone would not, however, explain why we failed to find a link between educational attainment and amygdala volume in the present study.

### Limitations

This study suffers from several limitations. First, by nature, it is difficult to draw strong conclusions concerning development and aging in a cross-sectional sample. While a longitudinal study spanning the entire 7-decade range of the present sample is unlikely to be feasible, longitudinal data are currently being acquired on a subset of these individuals, which may provide better evidence concerning rate of change.

Secondly, because this study represented a secondary analysis of an existing dataset, the sample was not originally recruited with the goal of optimizing educational diversity across the age spectrum. Thus, one limitation is the commonly reported negative correlation between age and education in the study participants (Piras et al., 2011). However, all analyses adjusted for age, and we attempted to mitigate such concerns in the interaction analyses by excluding overly influential points. Nonetheless, future investigations should intentionally recruit a sample in which age and education are fully de-confounded. Further, we do not have data regarding whether participants received all their education early in life, or whether they instead obtained part-time or “continuing education” later in adulthood.

Third, as mentioned above, we had no information on other aspects of objective or subjective SES, such as income, occupational status, or perceived social standing, which may have differentially predicted regional brain volumes. Nor did we have information on environmental factors, such as exposure to stressors or other putative mechanistic factors, which would have enabled us to directly test the degree to which such factors mediate our findings.

Fourth, although all participants were healthy at the time of testing, no specific screening for neurodegenerative disorders was performed, and it is therefore possible that some participants who would go on to develop neurodegenerative disease later on may have showed early signs of brain atrophy. A subset of participants had complete data on a memory recall and recognition test, and among this subset, scores were very similar to the full database of participants who completed this task, suggesting that at the time of scanning, the vast majority of participants had normal memory performance. Further, results were unchanged when known outliers on this were excluded. Nonetheless, this represents an important limitation given the increased risk of developing AD among individuals with low levels of education (Karp et al., 2004).

Finally, although the moderating effect of education on hippocampal volume across the lifespan is provocative, the direction of causality is unclear. Future work should build upon these findings by collecting longitudinal data on educational attainment, hypothesized environmental mediators, and brain structure over time.

## Conclusions

Across a 7-decade age range in adulthood, higher educational attainment buffers age-related decline in hippocampal volume, such that hippocampal volume tends to markedly decrease with age among less educated individuals, whereas hippocampal volumetric decreases with age are less pronounced among more educated individuals. No such effects were found for amygdala volume. This provides some support for “brain reserve theory” and suggests the possibility that higher education may be protective against age-related regional volumetric decreases. Alternatively, it is possible that higher education may allow for better compensatory mechanisms. Future research will be necessary to elucidate the mechanisms that underlie these effects.

### Conflict of interest statement

Dr. Grieve has received consulting fees from Brain Resource Ltd. Dr. Williams has received consulting fees and stock options in Brain Resource Ltd, and is a stock holder in Brain Resource Ltd. She has received advisory board fees from Pfizer. Other authors report no actual or potential commercial or financial relationships that could be construed as potential conflicts of interest.
